# Tall Fescue and *E. coenophiala* Genetics Influence Root-Associated Soil Fungi in a Temperate Grassland

**DOI:** 10.3389/fmicb.2019.02380

**Published:** 2019-10-15

**Authors:** Lindsey C. Slaughter, Jim A. Nelson, A. Elizabeth Carlisle, Marie Bourguignon, Randy D. Dinkins, Timothy D. Phillips, Rebecca L. McCulley

**Affiliations:** ^1^Department of Plant and Soil Sciences, University of Kentucky, Lexington, KY, United States; ^2^Department of Plant and Soil Science, Texas Tech University, Lubbock, TX, United States; ^3^USDA–ARS, Forage-Animal Production Research Unit, Lexington, KY, United States

**Keywords:** tripartite symbiosis, endophyte, grassland, *Neotyphodium*, plant-soil interactions

## Abstract

A constitutive, host-specific symbiosis exists between the aboveground fungal endophyte *Epichloë coenophiala* (Morgan-Jones & W. Gams) and the cool-season grass tall fescue (*Lolium arundinaceum* (Schreb.) Darbysh.), which is a common forage grass in the United States, Australia, New Zealand, and temperate European grasslands. New cultivars of tall fescue are continually developed to improve pasture productivity and animal health by manipulating both grass and *E. coenophiala* genetics, yet how these selected grass-endophyte combinations impact other microbial symbionts such as mycorrhizal and dark septate fungi remains unclear. Without better characterizing how genetically distinct grass-endophyte combinations interact with belowground microorganisms, we cannot determine how adoption of new *E. coenophiala*-symbiotic cultivars in pasture systems will influence long-term soil characteristics and ecosystem function. Here, we examined how *E. coenophiala* presence and host × endophyte genetic combinations control root colonization by belowground symbiotic fungi and associated plant nutrient concentrations and soil properties in a 2-year manipulative field experiment. We used four vegetative clone pairs of tall fescue that consisted of one endophyte-free (E−) and one *E. coenophiala*-symbiotic (E+) clone each, where E+ clones within each pair contained one of four endophyte genotypes: CTE14, CTE45, NTE16, or NTE19. After 2 years of growth in field plots, we measured root colonization of arbuscular mycorrhizal fungi (AMF) and dark septate endophytes (DSE), extraradical AMF hyphae in soil, total C, N, and P in root and shoot samples, as well as C and N in associated soils. Although we observed no effects of *E. coenophiala* presence or symbiotic genotype on total AMF or DSE colonization rates in roots, different grass-endophyte combinations altered AMF arbuscule presence and extraradical hyphal length in soil. The CTE45 genotype hosted the fewest AMF arbuscules regardless of endophyte presence, and E+ clones within NTE19 supported significantly greater soil extraradical hyphae compared to E− clones. Because AMF are often associated with improved soil physical characteristics and C sequestration, our results suggest that development and use of unique grass-endophyte combinations may cause divergent effects on long-term ecosystem properties.

## Introduction

Cool season grasses of the family Poaceae form the basis of many agricultural grasslands worldwide that are used to support grazing livestock, and substantially contribute to the over 178 million ha of pastureland in the US alone (United States Department of Agriculture National Agricultural Statistics service [USDA-NASS], n.d.). Many of these grasses naturally form host-specific symbioses with endophytic *Epichloë* spp. fungi (White, 1987; Leuchtmann, 1993; Clay and Schardl, 2002; Schardl et al., 2004). In the United States, researchers estimate that over 75% of the widespread perennial forage grass tall fescue (*Lolium arundinaceum* (Schreb.) Darbysh. = *Schedonorus arundinaceus* (Schreb.) Dumort = *Festuca arundinacea* Schreb.) naturally contains the asexual symbiotic endophyte *Epichloë coenophiala* (Morgan-Jones & W. Gams) [ = *Neotyphodium coenophialum* (Morgan-Jones & W. Gams) = *Acremonium coenophialum* Morgan-Jones & W. Gams] (Shelby and Dalrymple, 1987; Leuchtmann et al., 2014; Banfi et al., 2017). The interaction between tall fescue and *E. coenophiala* is a constitutive mutualistic symbiosis in which the asexual fungus is only transmitted vertically within seeds (Schardl et al., 2004). *E. coenophiala* can benefit its grass host through increased forage production, seed dispersal, drought tolerance, and protection from pathogen damage (Clay, 1988). One of the primary contributions to this symbiosis is that *E. coenophiala* can produce bioprotective compounds including ergot, loline, peramine, and indole diterpene alkaloids to deter mammalian and insect herbivory (Bush et al., 1997; Clay and Schardl, 2002). Yet, herbivore-deterring ergot alkaloids produced by the most common *E. coenophiala* strain found in the United States also causes toxicity symptoms such as reduced reproductive success and increased susceptibility to heat stress in grazing livestock (Schmidt and Osborn, 1993).

Many populations of tall fescue and related grasses in their native habitats across Europe and North Africa (Gibson and Newman, 2001) harbor asexual *Epichloë* strains that do not specifically produce these livestock-toxic ergot alkaloids, yet still defend hosts against insect herbivores and pathogens using similar alkaloid compounds such as lolines and peramine (Johnson et al., 2013). Researchers have isolated many of these *Epichloë* strains, now deemed non-toxic endophytes or novel endophytes (NTE), and inserted them into improved tall fescue cultivars for agricultural use to circumvent livestock stress and loss of cattle productivity caused by common toxic endophyte (CTE) strains (Bouton et al., 2002; Johnson et al., 2013). Although frequently classified as either CTE or NTE tall fescue, breeding efforts and natural selection have resulted in endophyte strains that differ in alkaloid production patterns, even within these CTE and NTE categories (Takach and Young, 2014; Young et al., 2014). Forage breeding programs have produced myriad genetically distinct grass-endophyte combinations, some of which eventually result in commercially available fescue seed. Examples include the combination of Jesup and GA-5 tall fescue cultivars containing the selected AR542 endophyte strain, commercially available as Jesup and GA-5 MaxQ tall fescue (Bouton et al., 2002), or insertion of the non-livestock toxic endophyte strain AR584 into Texoma tall fescue which created Texoma MaxQ II (Hopkins et al., 2011). Multiple additional cultivars of tall fescue containing selected NTE strains are commercially available and increasingly planted and evaluated for their use in forage livestock production (e.g., Kenyon et al., 2019), and researchers are continually developing new methodology to increase the efficiency of selecting and manipulating *E. coenophiala* strains to develop new grass-endophyte combinations (e.g., Ekanayake et al., 2017; Hettiarachchige et al., 2019).

Considering the large acreage of natural and agricultural land occupied by both NTE and CTE-symbiotic tall fescue (NTE+ and CTE+, respectively), these associations can have potentially large impacts on ecosystem properties and functions such as plant community dynamics, above- and belowground herbivore activities and soil food webs, and nutrient cycling (Rudgers and Clay, 2007; Omacini et al., 2012). For example, CTE+ tall fescue can steadily dominate plant communities or forage mixtures over time due to herbivore deterrence and enhanced competitive ability (Clay, 1996; Clay and Holah, 1999; Iqbal et al., 2013). Beyond its effects on aboveground plant communities, CTE+ tall fescue can reduce decomposition rates, increase C accumulation over time and alter soil microbial communities (Franzluebbers et al., 1999; Siegrist et al., 2010; Iqbal et al., 2012). Less is known about NTE+ tall fescue effects on plant communities and belowground dynamics. NTE+ tall fescue has demonstrated fewer negative effects on surrounding plant diversity than CTE+ tall fescue (Rudgers et al., 2010), but these effects are not easily generalizable between specific NTE+ strains and tall fescue cultivars (Yurkonis et al., 2014). Specific NTE genotypes also differ in their contributions to soil greenhouse gas emissions and C-cycling dynamics, as well as root exudate composition (Iqbal et al., 2013; Guo et al., 2015, 2016). These genetically distinct effects of different grass-endophyte combinations on both above- and belowground properties will likely have divergent long-term impacts on nutrient cycling and ecosystem productivity.

The underlying mechanisms that regulate how various CTE+ and NTE+ tall fescues impact belowground ecosystem properties have not been fully characterized. Potential drivers of these effects may include differences in root architecture and nutrient uptake dynamics (Malinowski and Belesky, 1999; Malinowski et al., 2000; Ding et al., 2016), and altered rhizosphere chemical profile differences between *E. coenophiala*-tall fescue associations referenced above (Guo et al., 2015, 2016). Another potential driver for belowground effects of *E. coenophiala* symbiosis in tall fescue are context-dependent interactions with other microorganisms within or on plant tissues. The myriad assemblages of bacteria and fungi in plants may or may not be host-specific and behave on a continuum from beneficial to negative outcomes for the host plant (Carroll, 1988; Johnson et al., 1997). For example, most land plants including tall fescue can form non-specific root symbioses with soil-borne fungi such as arbuscular mycorrhizal fungi (AMF) and dark septate endophytes (DSE) (Mandyam and Jumpponen, 2005; Smith and Read, 2008). The exact role and function of DSE is still unclear, although they can improve plant performance (Newsham, 2011). The role of AMF in plants is typically that of a nutritional mutualist, although the relative benefits of this association are context-dependent and vary based on plant and soil resource availability (Johnson et al., 1997). For example, AMF colonization is most beneficial to plant hosts under P-limitation despite some involvement in N-uptake, and varies based on plant functional or taxonomic group (Hoeksema et al., 2010). Commonly examined in agricultural ecosystems for their contributions to improved water and nutrient uptake in hosts (Augé, 2001) and enhancing long-term soil aggregation and C sequestration (Wilson et al., 2009), AMF may also play important roles in stress tolerance and pathogen defense (Barto et al., 2010; Abhiniti et al., 2013; Tao et al., 2016).

Prior work has shown that CTE+ tall fescue can reduce the presence of AMF propagules and spores (Chu-Chou et al., 1992), and lower abundance of the AMF-associated microbial lipid biomarker 16:1ω5cis in soil (Buyer et al., 2011). Colonization and subsequent sporulation of inoculated AMF can also be reduced in CTE+ tall fescue compared to *E. coenophiala*-free (E−) plants (Guo et al., 1992; Mack and Rudgers, 2008). Even in other grasses, addition of CTE+ tall fescue litter can inhibit AMF colonization compared to E− or NTE+ tall fescue (Antunes et al., 2008). Yet, the effects of both CTE+ and NTE+ tall fescue on AMF are inconsistent. Climate change factors such as warming and added precipitation can moderate the effects of distinct CTE and NTE strains in tall fescue on root symbionts such as AMF and DSE (Slaughter et al., 2018). In addition, some studies have found that neither CTE+ nor two genotypes of NTE+ tall fescue (AR542 and AR584) significantly affected root AMF colonization or extraradical hyphae in soil (Slaughter and McCulley, 2016; Kalosa-Kenyon et al., 2018), or the abundance of AMF lipid biomarkers in rhizosphere soil samples (Ding et al., 2016). In contrast, Rojas et al. (2016) found that E+ tall fescue regardless of CTE or NTE status increased relative abundance of the AMF phylum Glomeromycota via ITS1 rRNA sequencing but not the AMF lipid biomarker 16:1ω5cis in rhizosphere soil samples. Evaluation of other asexual *Epichloë* symbionts in cool-season grasses have similarly revealed negative (Müller, 2003; Liu et al., 2018), positive (Novas et al., 2005, 2009, 2011; Kazenel et al., 2015; Vignale et al., 2016), or mixed (Omacini et al., 2006; Liu et al., 2011; Larimer et al., 2012; Kalosa-Kenyon et al., 2018) effects on belowground AMF symbionts or microbial communities, with positive effects most prevalent in native or non-agronomic grasses. The inconsistencies described above highlight the need to better characterize the interactions between unique grass-endophyte combinations and belowground root symbionts, especially considering how new selected combinations are continually being developed and planted in agricultural grasslands.

Because both aboveground and belowground microbial associations are supported by photosynthetically produced plant C, tradeoffs likely exist in the capacity for a single host plant such as tall fescue to optimally benefit from, and meet the resource needs of, multiple mutualists. For example, previous studies have suggested that differing access to, or availability of, photosynthetically produced C (Mack and Rudgers, 2008) or competition between symbionts for plant C (Liu et al., 2011) may play an important role in mediating interactions between *Epichloë* endophytes and root fungi such as AMF or DSE. Because of its location in aerial tissues where plant C is initially fixed, as well as its initial presence in seeds rather than colonization from soil, Mack and Rudgers (2008) suggest that *E. coenophiala* may benefit from both spatial and temporal priority and thus exhibit greater competitive ability for plant resources compared to belowground symbionts. Both plant-fungal genetics and environmental conditions such as nutrient and moisture availability that regulate photosynthetic rates and C allocation between above- and belowground plant tissues are important context-dependent biotic and abiotic factors. These may, therefore, alter interactions between multiple fungal symbionts of grasses such as competition for plant resources, where *E. coenophiala* may have an advantage in accessing newly fixed plant C. All three of these symbionts are also typically regarded as having mutualistic interactions with host plants, a relationship that is likely to fluctuate between positive or negative outcomes across ecological contexts (Chamberlain et al., 2014). Therefore, we suggest considering this theoretical framework of biotic and abiotic context-dependency to delineate how evolutionarily coupled aboveground symbioses, such as between tall fescue and *E. coenophiala*, impact concurrent belowground associations with root-dwelling symbiotic fungi across different grass-endophyte combinations is necessary to determine how these agronomically selected plant-microbe interactions will impact future ecosystem functioning.

In this study, we examined how *E. coenophiala* presence and host × endophyte genetic combinations affect root colonization by belowground symbiotic fungi and associated plant nutrient concentrations and soil properties in a 2-year field experiment. We hypothesized that distinct *E. coenophiala* and tall fescue genotypic combinations would uniquely alter belowground colonization by AMF and DSE, potentially due to varying spatial and temporal priority effects or altered resource competition by different *E. coenophiala* strains. For example, limited plant C resources under drier conditions that reduce photosynthesis may promote antagonistic relationships between *E. coenophiala* and root symbionts, where *E. coenophiala* is better positioned to access C, compared to more synergistic relationships developing in resource-rich conditions where less symbiont competition is experienced. We expected the greatest reduction of AMF and DSE in CTE+ tall fescue due to greater competitive ability of CTE *E. coenophiala* genotypes for plant resources, and that this effect would be greatest under more stressful environmental conditions when symbiont antagonism might be most likely to occur.

## Materials and Methods

### Experimental Site and Study Design

This study took place within an existing long-term pasture climate change study at the University of Kentucky Spindletop Research Farm in Lexington, Kentucky (38°06′29.24″N; 84°29′29.72″W) as described in Slaughter et al. (2018) and Slaughter (2016). This site is 281 m above sea level, receives approximately 1163 mm annual precipitation (30-yr mean), and experiences mean annual summer and winter temperatures of 23.8°C and 1.6°C, respectively (Ferreira et al., 2010). The underlying soil is mapped as a Bluegrass-Maury silt loam complex with a 2% slope, which is a well-drained, fine-silty, mixed, active, mesic Typic Paleudalf formed from silty non-calcareous loess over clayey phosphatic limestone residuum (Soil Survey Staff, 2014).

The study pasture was established and seeded in spring 2008 with a mix of Kentucky bluegrass (*Poa pratensis* L.), tall fescue (*Lolium arundinaceum* (Schreb.) Darbysh.), red clover (*Trifolium pratense* L.), and white clover (*Trifolium repens* L.), with bermudagrass sprigs (*Cynodon dactylon* L. Pers.) plugged into the site in the fall. The experimental plots were established in 2009, with five blocks consisting of four, 5.8 m^2^ hexagonal plots randomly assigned to different climate manipulation treatments that consisted of factorial combinations of warming and added precipitation as described previously (McCulley et al., 2014; Slaughter et al., 2018). A concurrent study characterized the effects of warming (+Heat) and added precipitation (+Precip) using two of the grass-endophyte combinations also represented in this work (Slaughter et al., 2018). Of the 59 samples analyzed in Slaughter et al. (2018) from two genotypes and four climate treatments, 32 are shared with the current study, which included a total of 68 samples from four genotypes and two climate treatments in the statistical analysis. Samples analyzed in both the current study and Slaughter et al. (2018) thus included the “0 Heat, 0 Precip” (Control) and “0 Heat, +Precip” (+Precip) treatments only for the CTE45 and NTE19 symbiotic genotypes explained below. Because the +Heat treatments were stressful enough to induce substantial losses of two tall fescue-endophyte symbiotic combinations (CTE14 and NTE16, described below) and we were interested in the above- and belowground interactions of live symbiotic material, in this manuscript we evaluated the symbiotic material in the relatively unstressed mesic climate conditions only (Control and +Precip) where all four grass-endophyte genotypes survived. Plant materials used in both Slaughter et al. (2018) and the current study were planted and subjected to the same conditions and harvested at the same time as described below. Compared to the reduced number of grass-endophyte genotypes evaluated in Slaughter et al. (2018) (*n* = 2), this approach provided more symbiotic materials for comparison. In addition, evaluating mesic +Precip treatments and ambient Control conditions allowed us to assess how previously observed increases in photosynthesis rates and subsequent C availability due to added precipitation (Bourguignon et al., 2015) alters competitive interactions between fungal symbionts. We therefore analyzed samples for this study only from treatment plots that received either added precipitation (+Precip, + 30% of long-term annual mean precipitation added in growing season) or ambient environmental conditions (Control). Lateral water movement between treatments was restricted by installing aluminum flashing to a depth of 50 cm around each study plot, and additional water collected on site was applied to +Precip treatments only during existing precipitation events within the growing season (April–September). Soil moisture, air temperature, and soil temperature were continuously monitored in each plot throughout the study period from 2009 to 2013. As intended, +Precip plots typically exhibited up to 0.06 cm^–3^ cm^–3^ greater soil moisture on average than ambient controls across the study period, most notably in the summer and fall months, and more so in drier years, such as 2012, where +Precip plots were 0.07 cm^–3^ cm^–3^ higher than Controls in both May–June and August–September (Bourguignon et al., 2015). Soil moistures averaged 0.30 cm^–3^ cm^–3^ in +Precip plots and 0.26 cm^–3^ cm^–3^ in Control plots throughout the study period (McCulley et al., 2014; Bourguignon et al., 2015; Slaughter et al., 2018).

In October 2011, vegetative clone pairs of tall fescue (*n* = 4 pairs), where one individual of each clone was endophyte-infected with either a CTE or NTE strain and the other individual was treated with fungicide to be endophyte-free, were added to each experimental plot (*n* = 8 individuals planted per plot) within in a 30 × 60 cm area divided equally into 8 square sub-plots (one per plant), 4 of which (consisting of 4 plants from the total 8 per subplot in each climate treatment plot) were also referenced in Slaughter et al. (2018). The experimental design is further illustrated in Supplementary Figure S1. As described in Bourguignon et al. (2015), without disturbing the surrounding area or plants within each climate treatment, the total 8-square subplot within each climate treatment was treated with glyphosate [N-(phosphonomethyl)-glycine] prior to establishing clone pairs. The tall fescue plants established in subplots for this study were not excluded from neighboring plants within the whole-plot treatments using above or belowground barriers, but we did not observe significant plant encroachment from within the larger plots during the two study years. No fertilizer was applied throughout the study period. Plant biomass was harvested from each individual clone pair by clipping to a height of 6 cm three times per year during the study as described in Bourguignon et al. (2015). The clone pairs used for this study were selected from an assemblage developed in 2006 at the University of Kentucky by R. Dinkins, using seeds harvested from naturally CTE+ KY-31 tall fescue populations by T. Phillips. Endophyte-symbiotic individuals grown from seed were split into two parts, with half of the tillers treated with Folicur 3.6F fungicide (Bayer Crop Science, Monheim, Germany) [tebuconazole (1-[4-chlorophenyl]-4,4-dimethyl-3-[1,2,4-triazol-1-ylmethyl]pentan-3-ol)] to remove the CTE as described by Nagabhyru et al. (2013) and illustrated in Supplementary Figure S1 of Slaughter et al. (2018), thus creating E− vegetative clones of the CTE+ source material. Cloned NTE+/E− tall fescue pairs were similarly created in 2008 using NTE+ KY-31 tall fescue seeds developed by C. West (previously at University of Arkansas). Endophyte-free plants were only treated with fungicide once, during development, and all clones were transferred from initial greenhouse conditions to grow in test plots at the same United Kingdom research farm for several years before the current study. All material was periodically monitored for endophyte status as described previously (Nagabhyru et al., 2013; Bourguignon et al., 2015).

Because tall fescue reproduces both vegetatively and by obligate outcrossing to produce seeds, we consider E+/E− clone pairs of tall fescue developed vegetatively from a single seed to be genetically distinct from clone pairs developed from other seeds. Thus, we refer to different E+/E− clone pairs as different tall fescue genotypes. We selected E+/E− clones of four tall fescue genotypes for this study, two whose E+ clones associated with a CTE strain (genotypes 14 and 45), and two whose E+ clones associated with an NTE strain (genotypes 16 and 19). Because the *E. coenophiala* genotypes also differ between clone pairs of tall fescue, we use the term “tall fescue symbiotic genotype” to describe inseparable effects of tall fescue and *E. coenophiala* genotype in this study, or simply intend the genotype IDs (e.g., 14, 45, 16, 19) to interchangeably denote both the tall fescue and endophyte genotype within each grass-endophyte combination. The *E. coenophiala* genotypes within E+ individuals of each clone pair are commonly found both in existing United States grasslands or are available in commercial tall fescue cultivars as described in Young et al. (2014), where CTE genotypes 14 and 45 and NTE genotypes 16 and 19 are represented by *E. coenophiala* profiles 1, 2, 4, and FaTG-4, respectively.

### Sample Harvest and Preparation

Two years after planting, aboveground plant material, roots, and associated rhizosphere soil of each clone pair were harvested in October 2013 and stored at −20°C. Of the 80 individuals planted in 2011 (5 blocks × 2 treatment plots × 8 individuals per plot), 74 plants remained for harvest due to mortality during the 2-year period, during which three plants died in Control plots (one NTE19 E+, two NTE19 E−) and three plants died in +Precip plots (one CTE14 E−, one CTE14 E+, and one CTE45 E−). We separated aboveground plant material from roots and brushed all rhizosphere soil from the roots. Soils were sieved to 2 mm and air-dried. Washed and dried roots (48 h at 55°C) were weighed to determine root biomass, then ball-ground for nutrient measurements after subsections were used for mycorrhizal quantification. Plant shoots were dried (48 h at 55°C) and ball-ground for nutrient analyses. We corrected root and shoot weights for ash content using subsamples of ball-ground material combusted at 525°C for 4 h. We measured total C and N (%) in ball-ground sub-samples via combustion on an elemental analyzer (FlashEA 1112 series, Thermo Fisher Scientific, Waltham, MA, United States), as well as total P (%) via digestion (Fiske and Subbarow, 1925). Measured C, N, and P values were further used to calculate C:N ratio to determine differences in grass forage quality, where a lower C:N ratio results in higher forage quality, and N:P ratios, where N:P ratios >16 indicate relatively greater phosphorus limitation while N:P ratios <10 indicate greater nitrogen limitation (Güsewell, 2004).

### Verification of Endophyte Presence and Strain

Genetic profiling of *E. coenophiala* strains in each plant sample were conducted using DNA extraction and amplification of 11 fungal mating type, housekeeping, and biosynthesis genes across three multiplex PCR reactions as described previously (Takach and Young, 2014). Based on the presence and combinations of these 11 genes, we screened each plant sample for endophyte presence, including E− plants, and to verify that all E+ treatments contained the intended endophyte genotype (Supplementary Table S1 in Slaughter et al., 2018). Six of the total 74 samples collected tested opposite to expectations, where four E− samples tested as E+, one E+ sample tested E−, and one E+ sample harbored the incorrect treatment strain, and were thus excluded from statistical analysis.

### Root Fungal Colonization

Root fungal colonization was estimated in dried subsamples from each harvested tall fescue plant using trypan blue staining and microscopy (McGonigle et al., 1990) as described previously (Slaughter and McCulley, 2016; Slaughter et al., 2018). Roots were rehydrated and cleared in KOH, acidified with HCl, stained with 0.05% trypan blue solution, and de-stained in 1:1 glycerol: H_2_O. Ten 1 cm sub-sections were placed on microscope slides and viewed at 400x magnification. We counted trypan-stained AMF (arbuscules, vesicles, hyphae) and melanized DSE (microsclerotia, hyphae) structures using the line intersect method modified from McGonigle et al. (1990). Fungal colonization (%) was calculated as the number of presences divided by total views, then multiplied by 100.

### Extraradical AMF in Soil

We estimated the length of extraradical AMF hyphae (ERH) from soil samples using methods modified from Jakobsen et al. (1992) and Rillig et al. (1999) as described previously (Slaughter and McCulley, 2016; Slaughter et al., 2018). Fungal hyphae in dispersed soil samples were retained on a 38 μm sieve, re-suspended and stained with 0.05% trypan blue for 1.5 h (Brundrett et al., 1994), then vacuum-filtered through a 0.45 μm nitrocellulose membrane. Dried membrane filters were preserved and covered on 25 mm microscope slides using PVLG for microscopy analysis. We estimated the length of trypan-stained, non-septate or non-regularly septate ERH at 100× magnification within 50 fields of view on each slide using the gridline-intersect method described in Brundrett et al. (1994), with a 10 mm^2^ gridded graticular eyepiece (100 squares total), considering only hyphae that were trypan-stained and non-septate to be AMF. Hyphal length (m hyphae g^–1^ soil) was calculated using Tennant’s equation (Brundrett et al., 1994) and corrected for 89% extraction efficiency determined as in Miller et al. (1995).

### Statistical Analysis

The study used a split-split design within the established field plots, where whole plots (+Precip, Control) of the larger field study were split first by tall fescue symbiotic genotype clone pairs (CTE14, CTE45, NTE16, and NTE19), and then split by endophyte presence (E+) or absence (E−) within each clone pair. Measured fungal and plant nutrient response variables were analyzed for significant fixed effects (α = 0.05) of added precipitation (Precip), symbiotic genotype (TFtype), and endophyte status (Estatus) using PROC GLIMMIX in SAS (9.3 SAS Institute Inc., Cary, NC, United States). Arbuscule and vesicle data were arcsine-transformed and total shoot weight was log-transformed during analysis to meet statistical assumptions of normality, but untransformed values are shown in figures and tables where applicable to retain biological significance. Within significant interactions, we determined significant differences between means (α = 0.05) using Least Squares Means (LSMEANS/pdiff) in SAS. Complete ANOVA results for these analyses are included in Table 1 and Supplementary Tables S1–S3.

**TABLE 1:** Analysis of variance (ANOVA) results for the effects of independent treatment variables on belowground fungal parameters.

	**ERH**	**Total AMF**	**AMF arbuscules**	**AMF vesicles**	**AMF hyphae**	**DSE**
	
**Treatment effect**	**- m hyphae g^–1^ soil -**	**-% -**	**-% -**	**-% -**	**-% -**	**-% -**
Precip	*F*_1,4_^†^ = 0.02	*F*_1,4_ = 0.93	*F*_1,4_ = 1.20	*F*_1,4_ = 0.42	*F*_1,4_ = 0.04	*F*_1,4_ = 0.99
	*p* = 0.9026	*p* = 0.3891	*p* = 0.3344	*p* = 0.5508	p = 0.8591	*p* = 0.3759
TFtype	*F*_3,24_ = 0.66	*F*_3,24_ = 0.63	***F*_3,24_ = 5.46**	*F*_3,24_ = 0.65	*F*_3,24_ = 1.92	*F*_3,24_ = 0.75
	*p* = 0.5873	*p* = 0.6006	***p* = 0.0053**	*p* = 0.5917	*p* = 0.1538	*p* = 0.5339
TFtype × Precip	*F*_3,24_ = 0.07	*F*_3,24_ = 0.39	*F*_3,24_ = 1.27	*F*_3,24_ = 0.71	*F*_3,24_ = 1.95	*F*_3,24_ = 0.43
	*p* = 0.9753	*p* = 0.7602	*p* = 0.3085	*p* = 0.5568	*p* = 0.1492	*p* = 0.7367
Estatus	*F*_1,20_ = 0.58	*F*_1,20_ = 0.35	*F*_1,20_ = 0.59	***F*_1,20_ = 6.70**	*F*_1,20_ = 0.27	*F*_1,20_ = 2.30
	*p* = 0.4552	*p* = 0.5612	*p* = 0.4511	***p* = 0.0176**	*p* = 0.6079	*p* = 0.1451
Estatus × Precip	*F*_1,20_ = 0.32	*F*_1,20_ = 0.20	*F*_1,20_ = 0.26	*F*_1,20_ = 0.10	*F*_1,20_ = 0.08	*F*_1,20_ = 2.01
	*p* = 0.5774	*p* = 0.6593	*p* = 0.6182	*p* = 0.7607	*p* = 0.7783	*p* = 0.1719
TFtype × Estatus	***F*_3,20_ = 3.59**	*F*_3,20_ = 1.17	*F*_3,20_ = 1.06	*F*_3,20_ = 1.61	*F*_3,20_ = 1.04	*F*_3,20_ = 0.13
	***p* = 0.0319**	*p* = 0.3453	*p* = 0.3892	*p* = 0.2180	*p* = 0.3951	*p* = 0.9432
TFtype × Estatus × Precip	*F*_3,20_ = 0.92	*F*_3,20_ = 2.55	*F*_3,20_ = 0.92	*F*_3,20_ = 1.16	*F*_3,20_ = 2.65	*F*_3,20_ = 1.29
	*p* = 0.4480	*p* = 0.0850	*p* = 0.4478	*p* = 0.3503	*p* = 0.0769	*p* = 0.3044

*Significant *p*-values (α = 0.05) are denoted with bold type. ^†^Numerator, denominator degrees of freedom.*

## Results

### Root and Soil Fungi

**FIGURE 1:**
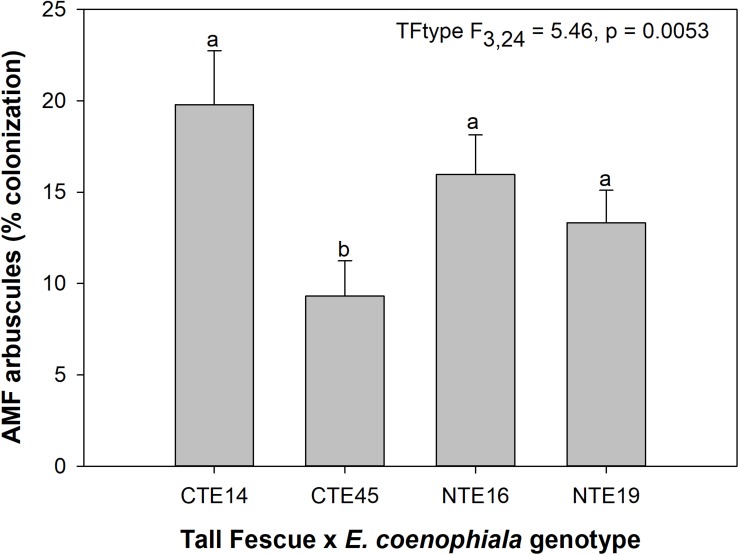
Main effect of tall fescue symbiotic genotype (TFtype) on arbuscule presence in tall fescue roots. Values are means ± 1 SE, and values with different letters are significantly different (α < 0.05).

Neither endophyte status nor tall fescue genotype significantly influenced total AMF colonization (arbuscules + vesicles + hyphae) in tall fescue roots. However, although not statistically significant (*p* = 0.0850), we observed that an interaction between tall fescue symbiotic genotype, endophyte status, and added precipitation marginally impacted total AMF colonization (Table 1). This was driven by differences occurring within +Precip plots, where endophyte-free clones of the grass-endophyte combination CTE45 (CTE45 E−) exhibited lower AMF colonization than NTE16 E− plants (42% and 63% colonization, respectively; *p* = 0.0348). CTE14 E− plants also had lower AMF colonization, with 43%, than NTE16 E− plants within +Precip plots, which was marginally significant (*p* = 0.0558). When averaged across both E− and E+ individuals, tall fescue symbiotic genotypes CTE14 and CTE45 expressed different colonization by AMF arbuscules, with CTE14 containing over twice the arbuscule colonization of CTE45 (Figure 1). In addition, AMF vesicle colonization was significantly higher in E− compared to E+ individuals across all tall fescue genotypes (3.28% ± 0.67 S.E. in E− vs. 1.71% ± 0.52 S.E. in E+; Table 1). Root colonization by DSE was not affected by tall fescue genotype, endophyte presence, or added precipitation (Table 1), averaging 10% across all study samples and ranging from an average of 6% in CTE45 E+ clones to 14% in NTE19 E− clones.

**FIGURE 2:**
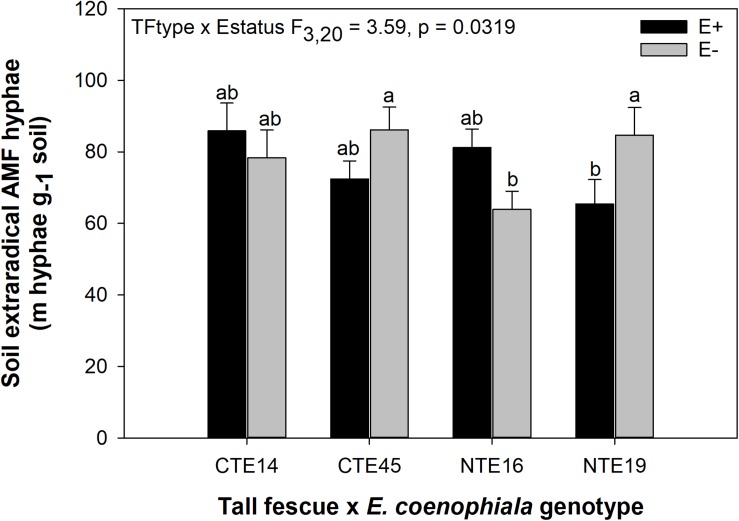
Interactive effects of tall fescue symbiotic genotype and endophyte status on the length of extraradical AMF hyphae (ERH) in root-associated soil. Bars indicate means ± 1 S.E. Bars sharing no common letter (a,b) indicate significant differences between means (α = 0.05).

ERH length in root-associated soils was influenced by both tall fescue genotype and endophyte status (Figure 2). ERH was significantly reduced in NTE16 E− clones compared to E− clones of either CTE45 or NTE19. In addition, NTE19 E− clones supported approximately 18 m hyphae g^–1^ soil more than NTE19 E+ clones (*p* = 0.0472), whereas E+ and E− levels were similar for the other symbiotic genotypes. Interestingly, ERH marginally increased (*p* = 0.0850) in NTE16 E+ clones compared to NTE16 E− clones, which was the opposite effect of that observed in NTE19.

### Plant Biomass and Nutrients

**FIGURE 3:**
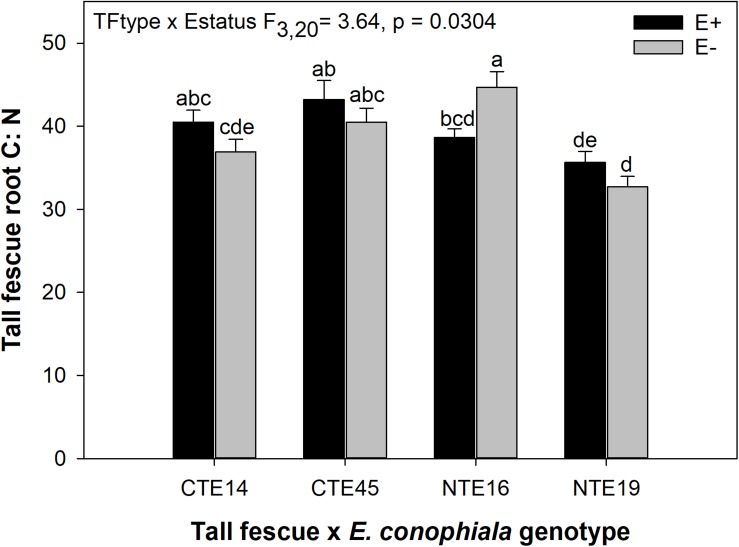
Interactive effects of tall fescue symbiotic genotype and endophyte status on the ratio of C:N in belowground tall fescue root tissue. Bars indicate means ± 1 S.E. Bars sharing no common letter (a,b,c,d,e) indicate significant differences between means (α = 0.05).

**FIGURE 4:**
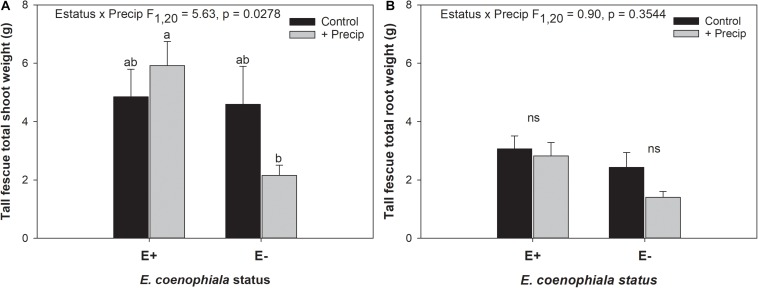
Interactive effects of endophyte status and added precipitation on total **(A)** shoot weight and **(B)** root weight in tall fescue. Bars indicate means ± 1 S.E. Within panel **(A)**, bars sharing no common letter (a,b) indicate significant differences between means (α = 0.05), Within panel **(B)**, lack of significant differences due to an interaction between endophyte status and added precipitation is indicated with “ns.”

Differences in tall fescue symbiotic genotypes, regardless of endophyte status, significantly impacted N%, N:P ratio and C:N ratio of tall fescue shoot tissue. Shoot N and N:P were significantly higher in symbiotic genotypes NTE16 and NTE19 compared to CTE14 and CTE45 (Table 2). Shoot C:N was highest in CTE14 and lowest in NTE19, yet also significantly differed between CTE14 vs. CTE45 and between NTE16 vs. NTE19 (Table 2). In addition, endophyte presence across all tall fescue symbiotic genotypes significantly stimulated shoot P% and root weight, while simultaneously decreasing both shoot and root N:P ratios in tall fescue tissue (Table 3). Both tall fescue symbiotic genotype and endophyte status interactively influenced C:N ratios of tall fescue roots (Figure 3). Specifically, tall fescue root C:N ratios were approximately 6 points less within E+ clones compared to E− clones of NTE16, but other genotypes had no differences between E+ and E− material. Endophyte status and added precipitation together influenced total shoot weight across all tall fescue symbiotic genotypes. With additional precipitation, endophyte effects became apparent, with E− fescue having significantly less shoot biomass than E+ clones regardless of symbiotic genotype (Figure 4A). We did not find a similar interactive effect of precipitation and endophyte status for root biomass (Figure 4B and Supplementary Table S3). However, root: shoot ratios were significantly altered by precipitation and endophyte status (*p* = 0.0401, Supplementary Table S3). Under added precipitation, root: shoot ratios were significantly lower in E+ clones than E− clones regardless of symbiotic genotype (0.49 ± 0.05 S.E. in E+ vs. 0.79 ± 0.10 in E−, respectively; *p* = 0.0313). Root: shoot ratios were similarly lowered by added precipitation among E+ clones, with 0.94 ± 0.16 S.E. in E+ without added precipitation and 0.49 ± 0.05 S.E. in E+ with added precipitation; *p* = 0.0364). Root: shoot ratios of E− clones without added precipitation (0.80 ± 0.12 S.E.) did not differ significantly from other endophyte status or precipitation combinations. In soils associated with tall fescue roots, we found no significant effects of endophyte status, tall fescue genotype, or added precipitation on total soil C or N (Supplementary Table S3).

**TABLE 2:** Main effect of tall fescue symbiotic genotype on plant nutrient characteristics in tall fescue tissue, where there were no interactions with endophyte status or precipitation.

	**Tall fescue × *E. coenophiala* genotype**				
		
	**CTE 14**	**CTE 45**	**NTE 16**	**NTE 19**	***p*-value**
Shoot N (%)	0.97 (0.03)^b^	0.98 (0.04)^b^	1.10 (0.04)^a^	1.19 (0.04)^a^	0.0001
Shoot N:P	3.43 (0.13)^b^	3.52 (0.13)^b^	4.10 (0.20)^a^	4.15 (0.19)^a^	0.0074
Shoot C:N	40.66 (1.26)^a^	36.47 (1.10)^b^	34.32 (1.14)^b^	30.26 (0.87)^c^	<0.0001

*Values are means ± 1 S.E. Across rows, means with different letters are significantly different (α = 0.05, *p*-value shown for main effect). Complete ANOVA results for all shoot nutrient parameters are provided in Supplementary Table S1. Interaction results for tissue nutrient or biomass parameters are presented in Figures 3, 4.*

**TABLE 3:** Main effect of endophyte status on nutrient characteristics and root biomass of tall fescue tissue.

	**Endophyte status**		
		
	**E−**	**E+**	***p*-value**
Shoot P (%)	0.22 (0.01)	0.24 (0.01)	0.0013
Shoot N:P	4.08 (0.13)	3.55 (0.11)	0.0031
Root N:P	4.80 (0.27)	3.96 (0.18)	0.0300
Root weight (g)	1.90 (0.28)	2.95 (0.31)	0.0129

*Values are means ± 1 S.E., and *p*-values are shown for significant differences across rows. Complete ANOVA results for all tissue nutrient and biomass parameters are provided in Supplementary Tables S1–S3. Interaction results for tissue nutrient or biomass parameters are presented in Figures 3, 4.*

## Discussion

Because *E. coenophiala* and tall fescue genotype interactions may differentially impact signaling and carbon allocation to belowground symbionts, we hypothesized that unique *E. coenophiala* and tall fescue genotypic combinations would elicit different belowground colonization by AMF and DSE, and that modifying plant photosynthate due to added water would alter these interactions. However, this was only partially supported by our study results. No effects of tall fescue and *E. coenophiala* genotype were observed on DSE colonization in tall fescue roots (Table 1). This contrasts with prior analysis in Slaughter et al. (2018) that considered only two of the four genotypes used in this study (CTE45 and NTE19) across four climate change treatments and found both significant endophyte symbiosis and warming effects, where DSE colonization was decreased in E+ samples but stimulated by warming. Such discrepancies indicate that the greater variety in CTE and NTE genotypic combinations used in this study created a more variable, less clear effect of *E. coenophiala* presence on DSE colonization, and demonstrates the importance of examining multi-symbiont interactions within multiple host genotypes.

The occurrence of AMF arbuscules was strongly controlled by tall fescue genotype regardless of *E. coenophiala* presence. Genotype CTE45 exhibited 4–10% less arbuscule colonization than the other three genotypes (Figure 1). Endophyte symbiosis significantly reduced the length of ERH in soils associated with NTE19, but had inconsistent effects in the other three genotypes (Figure 2). Yet, tall fescue genotype had little influence on AMF vesicles, although endophyte presence significantly reduced the occurrence of AMF vesicles. Surprisingly, none of these interactions were modified significantly by reduced shoot biomass and potentially less abundant photosynthate in E− clones due to added moisture (Figure 4A). Despite the large difference in biomass between E+ and E− clones under the+ Precip treatment, combined with previous findings that+ Precip reduced photosynthesis rates in E− clones (significant in NTE16; Bourguignon et al., 2015), this effect of added precipitation, and presumably reduced C availability, had no impact on belowground fungi or plant nutrients (Table 1 and Supplementary Tables S1–S3). This directly contradicts our hypothesis regarding increased symbiont antagonism under more C-limited conditions. Similarly, greater root biomass in E+ clones than in E− clones regardless of plant genotype (Table 3) may also have resulted in greater root C availability to belowground symbionts, yet belowground fungal colonization was not altered. Further, the lack of a clear relationship between belowground fungal measurements and plant nutrient or biomass parameters, despite significant endophyte- and tall fescue genotype effects on plant N and P characteristics (Tables 2, 3), suggests that plant and *E. coenophiala* genetics more heavily control plant nutrition at this site than belowground nutritional symbionts such as AMF. Previous studies have shown that *Epichloë* endophyte symbiosis and grass genotype alters P uptake, potentially due to root architecture or metabolic differences (Cheplick et al., 1989; Malinowski and Belesky, 1999). We also found increased shoot P in E+ plants, as well as increased shoot N:P in NTE genotypes compared to CTE regardless of endophyte status (Tables 2, 3), yet none of these effects were related to measured root fungal parameters.

Arbuscules are the primary nutrient-transfer interface between host and AMF symbiont, where exchange of photosynthetic plant C for nutrients acquired by AMF, such as N and P, occurs (Smith and Smith, 1989), and are considered a sign of vitality and active nutrient exchange between host and symbiont. As such, arbuscule presence can vary with time according to when nutrient uptake and transfer is demanded by the host plant (Mullen and Schmidt, 1993). Given the linkages between AMF colonization and plant nutrient status (e.g., Nouri et al., 2014), we expected to find a relationship specifically between arbuscules and plant nutrients such as N and P. Although we observed tall fescue genotypic differences in occurrence of arbuscules, there were no tall fescue genotype-specific effects on plant nutrients or biomass that might be related to the significant arbuscule reduction in CTE45. There is some evidence that both host and AMF genotypes may interact to determine presence, abundance, and morphology of different AMF structures such as arbuscules (Demuth et al., 1991; Smith and Smith, 1997). Our results suggest that some tall fescue genotypes, such as CTE45, are less inclined than others to form nutrient-transfer symbioses with AMF. This contradicts results in Slaughter et al. (2018), where analysis of only two of the four genotypes used in this study (CTE45 and NTE19) across four climate change treatments revealed significant effects of endophyte presence and added moisture, but no genotype effect on arbuscules. Our results in this study therefore highlight the importance of examining multi-symbiont relationships within a variety of host genotypes, as interactions may become more or less apparent depending on plant genetics and abiotic conditions.

AMF vesicles are thought to function as energy storage organs or as resting spores within or between root cortex cells (Smith and Read, 2008), yet little is known about what host or environmental characteristics specifically control vesicle production (Smith and Smith, 1997). Because endophyte presence reduced vesicle presence regardless of symbiotic genotype in this study, the mechanisms producing this effect must be related to characteristics shared by both CTE and NTE strains. Reidinger et al. (2012) found that total concentration of pyrrolizidine alkaloids (senecionine, seneciophylline, jacobine, jacozine and jacoline) was negatively related to AMF vesicle colonization in *Senecio jacobaea*. Because both CTE and NTE endophytes can usually produce loline alkaloids, it is tempting to suggest that presence of loline alkaloids such as N-formylloline, the dominant alkaloid produced by *E. coenophiala* (Bush et al., 1997), may have played a role in the endophyte-related reduction in AMF vesicle abundance observed in this study. However, of the four tall fescue genotypes examined, NTE16 does not produce loline alkaloids while the remaining three genotypes vary significantly in the total concentration of lolines produced [NTE19 > CTE14 > CTE45 (Bourguignon et al., 2015)]. Because the endophyte effect on vesicles was consistent across genotypes, loline alkaloid production was likely not the dominant causal factor of the response. In Antunes et al. (2008), presence of CTE+ tall fescue thatch stimulated vesicle production but decreased arbuscule colonization in *Bromus inermis* compared to E− thatch, suggesting that inoculated AMF were stressed by characteristics unique to CTE+ tall fescue, such as the presence of ergot alkaloids. These results contrast with our study, where vesicles decreased in response to endophyte presence regardless of strain. It is possible that differences in other non-loline alkaloids, or even other non-alkaloid metabolites, shared by both CTE and NTE strains were responsible for decreasing vesicle occurrence, or endophyte-related alteration of plant or mycorrhizal stress in plant tissues.

Some research suggests that AMF vesicles may be formed in response to stressful environmental conditions (Cooke et al., 1993; Smith and Read, 2008). If endophyte presence improved overall plant vigor and reduced plant or microbial stress, then a reduction in vesicle occurrence might be expected. Differences in plant shoot biomass due to endophyte status were only observed with increased moisture, which significantly reduced shoot biomass in E− plants (Figure 4A). This result is counter-intuitive because tall fescue typically prefers moist conditions, and the +Precip treatments increased growing season moisture as intended (Bourguignon et al., 2015; Slaughter et al., 2018). Alternative explanations for the reduction in E− shoot biomass with added moisture such as increased pest or disease pressure are possible, but we did not observe symptoms in the field or lab that might account for this effect. Our results are supported by a similar observation in Bourguignon et al. (2015), where lower photosynthesis rates in E− tall fescue clones were significantly exacerbated by+Precip treatments in NTE16 (Figure 2B in Bourguignon et al. (2015). Although endophyte effects were also observed on root and shoot N:P, shoot P, and root weight (Table 3), it is difficult to assess whether these effects indicate improved plant vigor subsequently reducing vesicles, especially given that we observed no strong correlations between vesicle colonization and plant nutrient or biomass measurements (Pearson *r* < 0.3 in all cases, not shown).

Consistent with our hypothesis, both tall fescue genotype and endophyte status influenced the length of ERH in mesic conditions. NTE16 had less ERH than CTE45 and NTE19, but only when endophyte-free. When E+, all symbiotic genotypes had similar ERH levels, which differed from our expectation that NTE symbioses would express intermediate effects compared to CTE and E−. These results contrast with prior studies that have reported a negative effect of CTE+ tall fescue on soil AMF either in terms of lipid biomarker abundance (Buyer et al., 2011), or through interfering with AMF colonization of neighboring plants, potentially through effects on soil hyphae (Antunes et al., 2008). We found significant differences between E+ and E− clones only in NTE19 (Figure 2). Inconsistencies in *E. coenophiala* effects on soil AMF also conflict with prior reports that E+ plants increased the relative abundance of *Glomeromycota* in soil regardless of endophyte genetics (Rojas et al., 2016), yet our study did not measure other AMF structures such as multinucleated spores in soil that could contribute to greater detection through sequencing methods (Viera and Glenn, 1990; Bécard and Pfeffer, 1993; Marleau et al., 2011). The fact that we only detected endophyte-symbiosis effects on ERH in NTE19 also suggests that production of ergot alkaloids, the predominantly recognized difference between CTE+ and NTE+ tall fescue, was not a causal factor in these results, as NTE19 does not produce ergot alkaloids (Bourguignon et al., 2015). Mummey and Rillig (2006) suggest that plants influence extraradical AMF in soils through allocation of C resources to AMF, changes in the rate of hyphal decomposition, or changes in active plant biomass present to support extraradical hyphae. However, we did not find consistent tall fescue genotype or endophyte status-mediated effects on plant nutrients or biomass that would help explain the mechanisms underlying these ERH results, nor were there any strong correlations between ERH and plant nutrient or biomass characteristics (Pearson *r* < 0.3 in all cases, not shown).

One reason that tall fescue genotype and endophyte presence significantly affected occurrence of specific AMF structures and abundance of ERH may have been differences in AMF species colonizing these plants, as different species exhibit different developmental and functional dynamics of AMF structures such as arbuscules and vesicles (Dodd et al., 2000). In addition, although AMF are often morphologically and functionally distinguished by their production of arbuscules and vesicles, these structures are not necessarily found in all AMF species (Smith and Smith, 1997). We were unable to distinguish between AMF species based on structures or to test whether AMF species differences were driving the trends in these data, but future studies should focus on characterizing how AMF community composition and morphological traits are altered due to *E. coenophiala* symbiosis and host-symbiont genetic variability in tall fescue. Overall, our results suggest that both endophyte symbiosis and tall fescue genotype influence AMF investment in different structures, such as arbuscules, vesicles, and extraradical hyphae. These may lead to divergent long-term responses in ecosystem processes such as nutrient cycling through alterations in presence and functioning of AMF structures such as arbuscules used for nutrient transfer. Although we did not observe immediate effects on soil C or N concentrations in this study, altered abundance of extraradical hyphae in soils could also lead to long term changes in C sequestration (Miller et al., 1995; Wilson et al., 2009; Duchicela et al., 2013).

The results of this study combined with other investigations of aboveground asexual *Epichloë* symbiosis in grasses on belowground fungal symbioses highlight the complexity of genetic and environmental controls on plant-microbe-soil interactions. Further, our measurements in this study were taken at one time point after 2 years of field growth even though these tripartite relationships are the result of dynamic processes that fluctuate with time and environmental conditions, which means we likely observed only a portion of these complex interactions. We recommend that future studies monitor interactions between these symbionts across multiple timepoints, and further suggest that more consideration be given to potential changes in community composition of root AMF and DSE communities, which may have been altered rather than total colonization but was not measured in this study. Still, our results suggest that differences in both plant and fungal genetics within constitutive symbioses, such as that between asexual *Epichloë* endophytes and cool-season grasses, may induce subtle shifts in belowground root symbiont associations that subsequently affect long-term ecosystem outcomes. These results potentially indicate that development of new grass-endophyte combinations and use in forage settings may have long-term impacts on soil properties and management. Forage breeders frequently manipulate plant-microbe symbioses to produce myriad genetically distinct grass-endophyte combinations from similar source materials, many of which have been successfully developed into commercial forage cultivars (Gundel et al., 2013; Johnson et al., 2013), yet the ecosystem consequences of these selected lines remain unknown. Our results and that of others clearly indicates that there is substantial diversity in the response of belowground fungal symbionts to grass host genetics and aboveground fungal endophyte strains. Given the prevalence of this symbiosis in nature and in human-constructed ecosystems (e.g., lawns), and the importance of belowground symbioses to ecosystem characteristics, a better understanding of the physiological mechanisms driving these interactions and ecosystem consequences is required.

## Conclusion

In this study, we found that although tall fescue symbiotic genotype and aboveground *E. coenophiala* symbiosis did not significantly alter total colonization by belowground AMF or DSE, they did affect the abundance of specific AMF structures such as arbuscules in roots and extraradical hyphae in soils. Tall fescue genotypes differed in their inclination to form nutritional symbioses with AMF, while *E. coenophiala* presence appeared to indirectly alleviate AMF stress, indicated by decreased vesicle production, potentially through stimulatory effects on tall fescue biomass or resource availability. Endophyte symbiosis significantly decreased the length of ERH in mesic conditions, perhaps through reduction in plant C allocated to AMF, but only in one tall fescue genotype. Here, we have demonstrated that both host-symbiont genetic variation and *E. coenophiala* symbiosis in tall fescue result in different plant and endophyte interactions with AMF, resulting in different AMF structures and potentially altering the functional role of these belowground symbionts. In this mesic environment, development of new commercial grass cultivars based on relatively small genetic differences in plants and constitutive aboveground fungal symbionts may have long-term effects on soil properties and ecosystem functioning.

## Data Availability Statement

The datasets generated for this study are available on request to the corresponding author.

## Author Contributions

LS and RM wrote the manuscript with input and support from all co-authors. LS performed the study measurements on harvested materials, processed the experimental data, and performed analyses. RM and JN conceived and established the long-term experimental pasture site. JN and AC maintained the study site, harvested study samples, and collected site-level study data. TP and RD created the tall fescue clone pairs. MB, RD, TP, JN, and AC established and maintained tall fescue clone pairs for the experiment.

## Conflict of Interest

The authors declare that the research was conducted in the absence of any commercial or financial relationships that could be construed as a potential conflict of interest.

## References

[B1] Abhiniti M. Tripti A. Trivedi P. (2013). Evaluation of AM fungi in management of meloidogyne-rhizoctonia complex infecting *Capsicum annuum* L. *Ann. Plant Prot. Sci.* 21 396–399.

[B2] Antunes P. M. Miller J. Carvalho L. M. Klironomos J. N. Newman J. A. (2008). Even after death the endophytic fungus of *Schedonorus phoenix* reduces the arbuscular mycorrhizas of other plants. *Funct. Ecol.* 22 912–918. 10.1111/j.1365-2435.2008.01432.x

[B3] Augé R. M. (2001). Water relations, drought and vesicular-arbuscular mycorrhizal symbiosis. *Mycorrhiza* 11 3–42. 10.1007/s005720100097

[B4] Banfi E. Galasso G. Foggi B. Kopecký D. Ardenghi N. M. G. (2017). From schedonorus and *Micropyropsis* to *Lolium* (Poaceae: Loliinae): new combinations and typifications. *TAXON* 66 708–717. 10.12705/663.11

[B5] Barto K. Friese C. Cipollini D. (2010). Arbuscular mycorrhizal fungi protect a native plant from allelopathic effects of an invader. *J. Chem. Ecol.* 36 351–360. 10.1007/s10886-010-9768-4 20229215

[B6] Bécard G. Pfeffer P. E. (1993). Status of nuclear division in arbuscular mycorrhizal fungi during in vitro development. *Protoplasma* 174 62–68. 10.1007/bf01404043

[B7] Bourguignon M. Nelson J. A. Carlisle E. Ji H. Dinkins R. D. Phillips T. D. (2015). Ecophysiological responses of tall fescue genotypes to fungal endophyte infection, elevated temperature, and precipitation. *Crop Sci.* 55 2895–2909.

[B8] Bouton J. H. Latch G. C. M. Hill N. S. Hoveland C. S. Mccann M. A. Watson R. H. (2002). Reinfection of tall fescue cultivars with non-ergot alkaloid–producing endophytes. *Agron. J.* 94 567–574.

[B9] Brundrett M. Addy H. Mcgonigle T. (1994). “Chapter 2: extracting, staining and measuring hyphae from soil,” in *Practical Methods in Mycorrhizal Research*, eds Brundrett M. Melville L. Peterson L. (Waterloo: Mycologue Publications Ltd.).

[B10] Bush L. P. Wilkinson H. H. Schardl C. L. (1997). Bioprotective alkaloids of grass-Fungal endophyte symbioses. *Plant Physiol.* 114 1–7. 10.1104/pp.114.1.1 12223685 PMC158272

[B11] Buyer J. S. Zuberer D. A. Nichols K. A. Franzluebbers A. J. (2011). Soil microbial community function, structure, and glomalin in response to tall fescue endophyte infection. *Plant Soil* 339 401–412. 10.1007/s11104-010-0592-y

[B12] Carroll G. (1988). Fungal endophytes in stems and leaves: from latent pathogen to mutualistic symbiont. *Ecology* 69 2–9. 10.2307/1943154

[B13] Chamberlain S. A. Bronstein J. L. Rudgers J. A. (2014). How context dependent are species interactions? *Ecol. Lett.* 17 881–890. 10.1111/ele.12279 24735225

[B14] Cheplick G. P. Clay K. Marks S. (1989). Interactions between infection by endophytic fungi and nutrient limitation in the grasses *Lolium perenne* and *Festuca arundinacea*. *New Phytol.* 111 89–97. 10.1111/j.1469-8137.1989.tb04222.x

[B15] Chu-Chou M. Guo B. An Z. Q. Hendrix J. W. Ferriss R. S. Siegel M. R. (1992). Suppression of mycorrhizal fungi in fescue by the *Acremonium coenophialum* endophyte. *Soil Biol. Biochem.* 24 633–637. 10.1016/0038-0717(92)90041-u

[B16] Clay K. (1988). Fungal endophytes of grasses: a defensive mutualism between plants and fungi. *Ecology* 69 10–16. 10.2307/1943155

[B17] Clay K. (1996). Interactions among fungal endophytes, grasses and herbivores. *Res. Popul. Ecol.* 38 191–201. 10.1007/bf02515727

[B18] Clay K. Holah J. (1999). Fungal endophyte symbiosis and plant diversity in successional fields. *Science* 285 1742–1744. 10.1126/science.285.5434.1742 10481011

[B19] Clay K. Schardl C. L. (2002). Evolutionary origins and ecological consequences of endophyte symbiosis with grasses. *Am. Nat.* 160 S99–S127. 10.1086/342161 18707456

[B20] Cooke M. A. Widden P. O’halloran I. (1993). Development of vesicular–arbuscular mycorrhizae in sugar maple (*Acer saccharum*) and effects of base-cation ammendment on vesicle and arbuscule formation. *Can. J. Bot.* 71 1421–1426. 10.1139/b93-171

[B21] Demuth K. Forstreuter W. Weber H. C. (1991). Morphological differences in vesicular-arbuscular mycorrhizae of gentianaceae produced by different endophytes. *Flora* 185 127–132. 10.1016/s0367-2530(17)30458-9

[B22] Ding N. Guo H. C. Kupper J. V. Mcnear D. H. (2016). Shoot specific fungal endophytes alter soil phosphorus (P) fractions and potential acid phosphatase activity but do not increase P uptake in tall fescue. *Plant Soil* 401 291–305. 10.1007/s11104-015-2757-1

[B23] Dodd J. C. Boddington C. L. Rodriguez A. Gonzalez-Chavez C. Mansur I. (2000). Mycelium of arbuscular mycorrhizal fungi (AMF) from different genera: form, function and detection. *Plant Soil* 226 131–151.

[B24] Duchicela J. Sullivan T. S. Bontti E. Bever J. D. (2013). Soil aggregate stability increase is strongly related to fungal community succession along an abandoned agricultural field chronosequence in the bolivian Altiplano. *J. Appl. Ecol.* 50 1266–1273.

[B25] Ekanayake P. N. Kaur J. Tian P. Rochfort S. J. Guthridge K. M. Sawbridge T. I. (2017). Genomic and metabolic characterisation of alkaloid biosynthesis by asexual Epichloe fungal endophytes of tall fescue pasture grasses. *Genome* 60 496–509. 10.1139/gen-2016-0173 28177829

[B26] Ferreira W. P. M. Priddy T. K. Souza C. F. Matthews J. (2010). “Trends in precipitation and air temperature time series in Lexington, KY-USA,” in *Proceedings of the ASABE, Annual International Meeting*, Pittsburgh, PA.

[B27] Fiske C. H. Subbarow Y. (1925). The colorimetric determination of phosphorus. *J. Biol. Chem.* 66 375–400.

[B28] Franzluebbers A. J. Nazih N. Stuedemann J. A. Fuhrmann J. J. Schomberg H. H. Hartel P. G. (1999). Soil carbon and nitrogen pools under low- and high-endophyte-infected tall fescue. *Soil Sci. Soc. Am. J.* 63 1687–1694.

[B29] Gibson D. J. Newman J. A. (2001). Festuca arundinacea Schreber (F. elatior L. ssp. arundinacea (Schreber) Hackel). *J. Ecol.* 89 304–324. 10.1046/j.1365-2745.2001.00561.x

[B30] Gundel P. E. Pérez L. I. Helander M. Saikkonen K. (2013). Symbiotically modified organisms: nontoxic fungal endophytes in grasses. *Trends Plant Sci.* 18 420–427. 10.1016/j.tplants.2013.03.003 23562460

[B31] Guo B. Z. Hendrix J. W. An Z. Q. Ferriss R. S. (1992). Role of *Acremonium* endophyte of fescue on inhibition of colonization and reproduction of mycorrhizal fungi. *Mycologia* 84 882–885. 10.1080/00275514.1992.12026220

[B32] Guo J. Mcculley R. L. Mcnear D. H. (2015). Tall fescue cultivar and fungal endophyte combinations influence plant growth and root exudate composition. *Front. Plant Sci.* 6:183. 10.3389/fpls.2015.00183 25914697 PMC4391242

[B33] Guo J. Mcculley R. L. Phillips T. D. Mcnear D. H. (2016). Fungal endophyte and tall fescue cultivar interact to differentially affect bulk and rhizosphere soil processes governing C and N cycling. *Soil Biol. Biochem.* 101 165–174. 10.1016/j.soilbio.2016.07.014

[B34] Güsewell S. (2004). N : P ratios in terrestrial plants: variation and functional significance. *New Phytol.* 164 243–266. 10.1002/ece3.2587 33873556

[B35] Hettiarachchige I. K. Elkins A. C. Reddy P. Mann R. C. Guthridge K. M. Sawbridge T. I. (2019). Genetic modification of asexual Epichloe endophytes with the perA gene for peramine biosynthesis. *Mol. Genet Genomics* 294 315–328. 10.1007/s00438-018-1510-x 30443676

[B36] Hoeksema J. D. Chaudhary V. B. Gehring C. A. Johnson N. C. Karst J. Koide R. T. (2010). A meta-analysis of context-dependency in plant response to inoculation with mycorrhizal fungi. *Ecol. Lett.* 13 394–407. 10.1111/j.1461-0248.2009.01430.x 20100237

[B37] Hopkins A. A. Young C. A. Butler T. J. Bouton J. H. (2011). Registration of ‘Texoma’. MaxQ II tall fescue. *J. Plant Regist.* 5 14–18.

[B38] Iqbal J. Nelson J. A. Mcculley R. L. (2013). Fungal endophyte presence and genotype affect plant diversity and soil-to-atmosphere trace gas fluxes. *Plant Soil* 364 15–27. 10.1007/s11104-012-1326-0

[B39] Iqbal J. Siegrist J. A. Nelson J. A. Mcculley R. L. (2012). Fungal endophyte infection increases carbon sequestration potential of southeastern USA tall fescue stands. *Soil Biol. Biochem.* 44 81–92. 10.1016/j.soilbio.2011.09.010

[B40] Jakobsen I. Abbott L. K. Robson A. D. (1992). External hyphae of vesicular-arbuscular mycorrhizal fungi associated with *Trifolium subterraneum* L. *New Phytol.* 120 371–380. 10.1111/j.1469-8137.1992.tb01077.x

[B41] Johnson L. J. De Bonth A. C. M. Briggs L. R. Caradus J. R. Finch S. C. Fleetwood D. J. (2013). The exploitation of epichloae endophytes for agricultural benefit. *Fungal Divers.* 60 171–188. 10.1007/s13225-013-0239-4

[B42] Johnson N. C. Graham J. H. Smith F. A. (1997). Functioning of mycorrhizal associations along the mutualism–parasitism continuum. *New Phytol.* 135 575–585. 10.1046/j.1469-8137.1997.00729.x 16305138

[B43] Kalosa-Kenyon E. Slaughter L. C. Rudgers J. A. Mcculley R. L. (2018). Asexual *Epichloë* endophytes do not consistently alter arbuscular mycorrhizal fungi colonization in three grasses. *Am. Midl. Nat.* 179 157–165. 10.1674/0003-0031-179.2.157

[B44] Kazenel M. R. Debban C. L. Ranelli L. Hendricks W. Q. Chung Y. A. Pendergast T. H. (2015). A mutualistic endophyte alters the niche dimensions of its host plant. *AoB Plants* 7 lv005. 10.1093/aobpla/plv005 25603965 PMC4354242

[B45] Kenyon S. L. Roberts C. A. Lory J. A. Bailey E. A. Kallenbach R. L. Rottinghaus G. E. (2019). Comparison and diet preference of novel endophyte-infected tall fescue cultivars. *Crop Sci.* 59 1317–1329.

[B46] Larimer A. L. Bever J. D. Clay K. (2012). Consequences of simultaneous interactions of fungal endophytes and arbuscular mycorrhizal fungi with a shared host grass. *Oikos* 121 2090–2096. 10.1111/j.1600-0706.2012.20153.x

[B47] Leuchtmann A. (1993). Systematics, distribution, and host specificity of grass endophytes. *Nat. Toxins* 1 150–162. 10.1002/nt.2620010303 1344916

[B48] Leuchtmann A. Bacon C. W. Schardl C. L. White J. F. Jr. Tadych M. (2014). Nomenclatural realignment of *Neotyphodium* species with genus *Epichloe*. *Mycologia* 106 202–215. 10.3852/106.2.202 24459125

[B49] Liu H. Wu M. Ren A.-Z. Gao Y.-B. (2018). Effects of *Epichloe* endophytes of *Achnatherum* sibiricum on spore germination of arbuscular mycorrhizal fungi. *Ying Yong Sheng tai Xue Bao* 29 4145–4151. 30584743 10.13287/j.1001-9332.201812.040

[B50] Liu Q. Parsons A. J. Xue H. Fraser K. Ryan G. D. Newman J. A. (2011). Competition between foliar *Neotyphodium* lolii endophytes and mycorrhizal glomus spp. fungi in *Lolium perenne* depends on resource supply and host carbohydrate content. *Funct. Ecol.* 25 910–920. 10.1111/j.1365-2435.2011.01853.x

[B51] Mack K. M. L. Rudgers J. A. (2008). Balancing multiple mutualists: asymmetric interactions among plants, arbuscular mycorrhizal fungi, and fungal endophytes. *Oikos* 117 310–320. 10.1111/j.2007.0030-1299.15973.x

[B52] Malinowski D. P. Alloush G. A. Belesky D. P. (2000). Leaf endophyte *Neotyphodium coenophialum* modifies mineral uptake in tall fescue. *Plant Soil* 227 115–126.

[B53] Malinowski D. P. Belesky D. P. (1999). *Neotyphodium coenophialum*-endophyte infection affects the ability of tall fescue to use sparingly available phosphorus. *J. Plant Nutr.* 22 835–853. 10.1080/01904169909365675

[B54] Mandyam K. Jumpponen A. (2005). Seeking the elusive function of the root-colonising dark septate endophytic fungi. *Stud. Mycol.* 53 173–189. 10.3114/sim.53.1.173

[B55] Marleau J. Dalpe Y. St-Arnaud M. Hijri M. (2011). Spore development and nuclear inheritance in arbuscular mycorrhizal fungi. *BMC Evol. Biol.* 11:11. 10.1186/1471-2148-11-51 21349193 PMC3060866

[B56] McCulley R. L. Bush L. P. Carlisle A. E. Ji H. Nelson J. A. (2014). Warming reduces tall fescue abundance but stimulates toxic alkaloid concentrations in transition zone pastures of the U.S. *Front. Chem.* 2:88. 10.3389/fchem.2014.00088 25374886 PMC4204602

[B57] McGonigle T. P. Miller M. H. Evans D. G. Fairchild G. L. Swan J. A. (1990). A new method which gives an objective measure of colonization of roots by vesicular—arbuscular mycorrhizal fungi. *New Phytol.* 115 495–501. 10.1111/j.1469-8137.1990.tb00476.x 33874272

[B58] Miller R. M. Jastrow J. D. Reinhardt D. R. (1995). External hyphal production of vesicular-arbuscular mycorrhizal fungi in pasture and tallgrass prairie communities. *Oecologia* 103 17–23. 10.1007/BF00328420 28306940

[B59] Mullen R. B. Schmidt S. K. (1993). Mycorrhizal infection, phosphorus uptake, and phenology in *Ranunculus* adoneus: implications for the functioning of mycorrhizae in alpine systems. *Oecologia* 94 229–234. 10.1007/BF00341321 28314036

[B60] Müller J. (2003). Artificial infection by endophytes affects growth and mycorrhizal colonisation of *Lolium perenne*. *Funct. Plant Biol.* 30 419–424. 10.1071/FP02189 32689026

[B61] Mummey D. L. Rillig M. C. (2006). The invasive plant species *Centaurea maculosa* alters arbuscular mycorrhizal fungal communities in the field. *Plant Soil* 288 81–90. 10.1007/s11104-006-9091-6

[B62] Nagabhyru P. Dinkins R. D. Wood C. L. Bacon C. W. Schardl C. L. (2013). Tall fescue endophyte effects on tolerance to water-deficit stress. *BMC Plant Biol.* 13:127. 10.1186/1471-2229-13-127 24015904 PMC3848598

[B63] Newsham K. K. (2011). A meta-analysis of plant responses to dark septate root endophytes. *New Phytol.* 190 783–793. 10.1111/j.1469-8137.2010.03611.x 21244432

[B64] Nouri E. Breuillin-Sessoms F. Feller U. Reinhardt D. (2014). Phosphorus and nitrogen regulate arbuscular mycorrhizal symbiosis in *Petunia hybrida*. *PLoS One* 9:e90841. 10.1371/journal.pone.0090841 24608923 PMC3946601

[B65] Novas M. V. Cabral D. Godeas A. M. (2005). Interaction between grass endophytes and mycorrhizas in *Bromus setifolius* from Patagonia. *Argent. Symb.* 40 23–30.

[B66] Novas M. V. Iannone L. J. Godeas A. M. Cabral D. (2009). Positive association between mycorrhiza and foliar endophytes in *Poa bonariensis*, a native grass. *Mycol. Prog.* 8 75–81. 10.1007/s11557-008-0579-8

[B67] Novas M. V. Iannone L. J. Godeas A. M. Scervino J. M. (2011). Evidence for leaf endophyte regulation of root symbionts: effect of *Neotyphodium* endophytes on the pre-infective state of mycorrhizal fungi. *Symbiosis* 55 19–28. 10.1007/s13199-011-0140-4

[B68] Omacini M. Eggers T. Bonkowski M. Gange A. C. Jones T. H. (2006). Leaf endophytes affect mycorrhizal status and growth of co-infected and neighbouring plants. *Funct. Ecol.* 20 226–232. 10.1111/j.1365-2435.2006.01099.x

[B69] Omacini M. Semmartin M. Pérez L. I. Gundel P. E. (2012). Grass–endophyte symbiosis: a neglected aboveground interaction with multiple belowground consequences. *Appl. Soil Ecol.* 61 273–279. 10.1016/j.apsoil.2011.10.012

[B70] Reidinger S. Eschen R. Gange A. C. Finch P. Bezemer T. M. (2012). Arbuscular mycorrhizal colonization, plant chemistry, and aboveground herbivory on Senecio jacobaea. *Acta Oecol.* 38 8–16. 10.1016/j.actao.2011.08.003

[B71] Rillig M. C. Field C. B. Allen M. F. (1999). Soil biota responses to long-term atmospheric CO2 enrichment in two California annual grasslands. *Oecologia* 119 572–577. 10.1007/s004420050821 28307716

[B72] Rojas X. Guo J. Leff J. W. Mcnear D. H. Fierer N. Mcculley R. L. (2016). Infection with a shoot-specific fungal endophyte (*Epichloë*) alters tall fescue soil microbial communities. *Microb. Ecol.* 72 197–206. 10.1007/s00248-016-0750-8 26992401

[B73] Rudgers J. A. Clay K. (2007). Endophyte symbiosis with tall fescue: how strong are the impacts on communities and ecosystems? *Fungal Biol. Rev.* 21 107–124. 10.1016/j.fbr.2007.05.002

[B74] Rudgers J. A. Fischer S. Clay K. (2010). Managing plant symbiosis: fungal endophyte genotype alters plant community composition. *J. Appl. Ecol.* 47 468–477. 10.1111/j.1365-2664.2010.01788.x

[B75] Schardl C. L. Leuchtmann A. Spiering M. J. (2004). Symbioses of grasses with seedborne fungal endophytes. *Annu. Rev. Plant Biol.* 55 315–340. 10.1146/annurev.arplant.55.031903.141735 15377223

[B76] Schmidt S. P. Osborn T. G. (1993). Effects of endophyte-infected tall fescue on animal performance. *Agric. Ecosyst. Environ.* 44 233–262. 10.1016/0167-8809(93)90049-u

[B77] Shelby R. A. Dalrymple L. W. (1987). Incidence and distribution of the tall fescue endophyte in the United States. *Plant Dis.* 71 783–786.

[B78] Siegrist J. A. Mcculley R. L. Bush L. P. Phillips T. D. (2010). Alkaloids may not be responsible for endophyte-associated reductions in tall fescue decomposition rates. *Funct. Ecol.* 24 460–468. 10.1111/j.1365-2435.2009.01649.x

[B79] Slaughter L. C. (2016). *Effects of Epichloë Coenophiala-Tall Fescue Symbiosis on Plant-Microbe-Soil Interactions in a Temperate Pasture.* Ph.D. Theses, University of Kentucky, Lexington, KY.

[B80] Slaughter L. C. McCulley R. L. (2016). Aboveground *Epichloë coenophiala*-grass associations do not affect belowground fungal symbionts or associated plant, soil parameters. *Microb. Ecol.* 72 682–691. 10.1007/s00248-016-0828-3 27502203

[B81] Slaughter L. C. Nelson J. A. Carlisle E. Bourguignon M. Dinkins R. D. Phillips T. D. (2018). Climate change and *Epichloë coenophiala* association modify belowground fungal symbioses of tall fescue host. *Fungal Ecol.* 31 37–46. 10.1016/j.funeco.2017.10.002

[B82] Smith F. Smith S. (1989). Membrane transport at the biotrophic interface: an overview. *Funct. Plant Biol.* 16 33–43.

[B83] Smith F. A. Smith S. E. (1997). Tansley Review No. 96 Structural diversity in (vesicular)-arbuscular mycorrhizal symbioses. *New Phytol.* 137 373–388. 10.1046/j.1469-8137.1997.00848.x 33863081

[B84] Smith S. E. Read D. J. (2008). *Mycorrhizal Symbiosis.* London, UK: Academic Press.

[B85] Soil Survey Staff (2014). *Web Soil Survey Natural Resources Conservation Service, United States Department of Agriculture.* Available at: http://websoilsurvey.nrcs.usda.gov/ (accessed 9 October 2015).

[B86] Takach J. E. Young C. A. (2014). Alkaloid genotype diversity of tall fescue endophytes. *Crop Sci.* 54 667–678.

[B87] Tao L. Ahmad A. De Roode J. C. Hunter M. D. (2016). Arbuscular mycorrhizal fungi affect plant tolerance and chemical defences to herbivory through different mechanisms. *J. Ecol.* 104 561–571. 10.1111/1365-2745.12535

[B88] United States Department of Agriculture National Agricultural Statistics service [USDA-NASS] (n.d.). Available: https://quickstats.nass.usda.gov/results/FDBB91E2-. (2234)-323C-A285-FDF17F9C3669 (accessed May 8 2019).

[B89] Viera A. Glenn M. G. (1990). DNA content of vesicular-arbuscular mycorrhizal fungal spores. *Mycologia* 82 263–267. 10.1080/00275514.1990.12025874

[B90] Vignale M. V. Iannone L. J. Pinget A. D. De Battista J. P. Novas M. V. (2016). Effect of epichloid endophytes and soil fertilization on arbuscular mycorrhizal colonization of a wild grass. *Plant Soil* 405 279–287. 10.1007/s11104-015-2522-5

[B91] White J. Jr. (1987). Widespread distribution of endophytes in the Poaceae. *Plant Dis.* 71 340–342.

[B92] Wilson G. W. T. Rice C. W. Rillig M. C. Springer A. Hartnett D. C. (2009). Soil aggregation and carbon sequestration are tightly correlated with the abundance of arbuscular mycorrhizal fungi: results from long-term field experiments. *Ecol. Lett.* 12 452–461. 10.1111/j.1461-0248.2009.01303.x 19320689

[B93] Young C. A. Charlton N. D. Takach J. E. Swoboda G. A. Trammell M. A. Huhman D. V. (2014). Characterization of *Epichloe coenophiala* within the US: are all tall fescue endophytes created equal? *Front. Chem.* 2:95. 10.3389/fchem.2014.00095 25408942 PMC4219521

[B94] Yurkonis K. A. Shukla K. Holdenried J. Hager H. A. Bolton K. A. Klironomos J. N. (2014). Endophytes inconsistently affect plant communities across *Schedonorus arundinaceus* hosts. *Plant Ecol.* 215 389–398. 10.1007/s11258-014-0309-z

