# Supplementary material for: Tall Fescue and E. coenophiala Genetics Influence Root-Associated Soil Fungi in a Temperate Grassland
Source: Front Microbiol. 2019 Oct 15;10:2380. doi: 10.3389/fmicb.2019.02380 (PMC6843077; doi:10.3389/fmicb.2019.02380)
Supplement: Supplementary file 1 [file Table_1.docx]

Supplementary Material

# Supplementary Figures


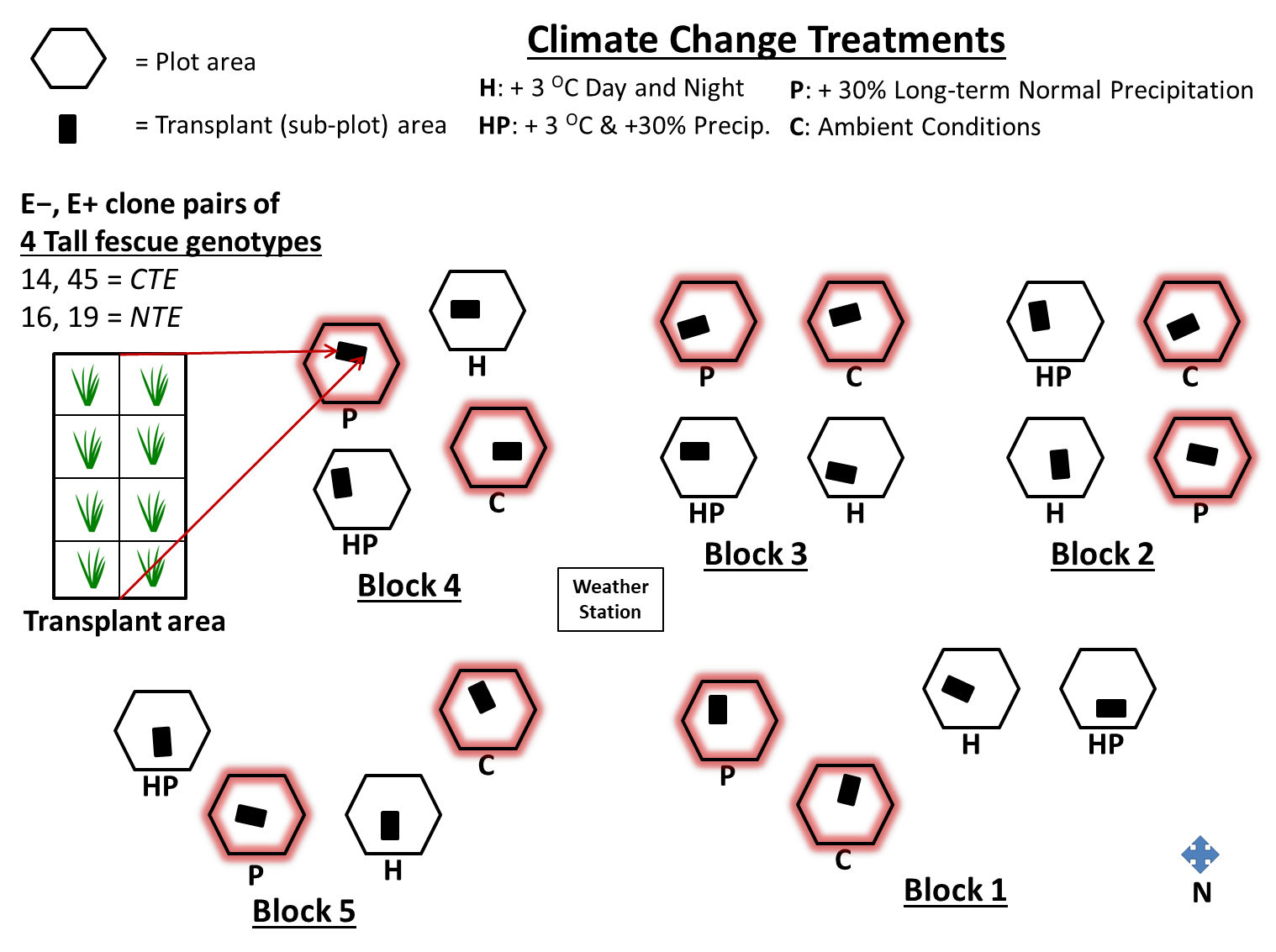


**Figure S1:** Experimental design of the long-term field climate change project established in 2008 at the UK Spindletop research farm in Lexington, KY. Factorial combinations of added heat (H), and added precipitation (P) were applied to hexagonal, 5.8 m^2^ plots (C = control/ ambient climate conditions). Cloned pairs of four tall fescue genotypes, where one clone was endophyte-infected (E+) and one clone was endophyte-free (E−) were transplanted into each climate treatment plot in 2011 (n = 8 plants per plot). E+ clones of tall fescue genotypes 14 and 45 contained common toxic endophyte (CTE) strains, whereas E+ clones of tall fescue genotypes 16 and 19 contained non-toxic endophyte (NTE) strains. All four grass-endophyte genotypes within the transplant area, but only from the mesic climate treatment plots highlighted in red, were used for analysis in this study. Data collected from two grass-endophyte genotypes (specifically C and P samples of genotypes CTE45 and NTE19; n=32 samples out of the n=68 analyzed here) that were previously published in Slaughter et al. (2018) were used in the analysis presented in this manuscript.

# Supplementary Tables

**Table S1:** Analysis of variance (ANOVA) results for the effects of independent treatment variables on shoot nutrient parameters. Significant p-values (α = 0.05) are denoted with bold type.

|  | **Shoot P** | **Shoot N** | **Shoot C** | **Shoot N:P** | **Shoot C:N** |
| --- | --- | --- | --- | --- | --- |
| Treatment Effect | **- % -** | **- % -** | **- % -** | **--** | **--** |
| **Precip** | F_1,4_^†^ = 0.05  p = 0.8385 | F_1,4_ = 3.88  p = 0.1201 | F_1,4_ = 0.67  p = 0.4575 | F_1,4_ = 3.18  p = 0.1493 | F_1,4_ = 5.79  p = 0.0738 |
|  |  |  |  |  |  |
| **TFtype** | F_3,24_ = 1.08  p = 0.3758 | **F_3,24_ = 10.93**  **p = 0.0001** | F_3,24_ = 2.27  p = 0.1063 | **F_3,24_ = 5.06**  **p = 0.0074** | **F_3,24_ = 17.32**  **p <0.0001** |
|  |  |  |  |  |  |
| **TFtype x Precip** | F_3,24_ = 1.24  p = 0.3176 | F_3,24_ = 0.15  p = 0.9284 | F_3,24_ = 0.28  p = 0.8386 | F_3,24_ = 0.71  p = 0.5528 | F_3,24_ = 0.23  p = 0.8752 |
|  |  |  |  |  |  |
| **Estatus** | **F_1,20_ = 14.08**  **p = 0.0013** | F_1,20_ = 0.80  p = 0.3808 | F_1,20_ = 0.63  p = 0.4357 | **F_1,20_ = 11.34**  **p = 0.0031** | F_1,20_ = 0.02  p = 0.8818 |
|  |  |  |  |  |  |
| **Estatus x Precip** | F_1,20_ = 0.10  p = 0.7493 | F_1,20_ = 0.26  p = 0.6180 | F_1,20_ = 0.94  p = 0.3446 | F_1,20_ = 0.90  p = 0.3528 | F_1,20_ = 1.48  p = 0.2383 |
|  |  |  |  |  |  |
| **TFtype x Estatus** | F_3,20_ = 1.09  p = 0.3742 | F_3,20_ = 0.68  p = 0.5717 | F_3,20_ = 0.46  p = 0.7133 | F_3,20_ = 0.23  p = 0.8758 | F_3,20_ = 0.88  p = 0.4687 |
|  |  |  |  |  |  |
| **TFtype x Estatus x Precip** | F_3,20_ = 1.00  p = 0.4124 | F_3,20_ = 0.42  p = 0.7375 | F_3,20_ = 0.84  p = 0.4877 | F_3,20_ = 0.79  p = 0.5122 | F_3,20_ = 0.96  p = 0.4308 |

† Numerator, denominator degrees of freedom

**Table S2:** Analysis of variance (ANOVA) results for the effects of independent treatment variables on root nutrient parameters. Significant p-values (α = 0.05) are denoted with bold type.

|  | **Root P** | **Root N** | **Root C** | **Root N:P** | **Root C:N** |
| --- | --- | --- | --- | --- | --- |
| Treatment Effect | **- % -** | **- % -** | **- % -** | **--** | **--** |
| **Precip** | F_1,4_^†^ = 0.26  p = 0.6373 | F_1,4_ = 0.92  p = 0.3908 | F_1,4_ = 0.59  p = 0.4837 | F_1,4_ = 0.74  p = 0.4389 | F_1,4_ = 0.03  p = 0.8632 |
|  |  |  |  |  |  |
| **TFtype** | F_3,24_ = 1.26  p = 0.3092 | F_3,24_ = 1.00  p = 0.4102 | F_3,24_ = 1.36  p = 0.2784 | F_3,24_ = 0.54  p = 0.6576 | **F_3,24_ = 9.37**  **p = 0.0003** |
|  |  |  |  |  |  |
| **TFtype x Precip** | F_3,24_ = 0.71  p = 0.5561 | F_3,24_ = 0.61  p = 0.6137 | F_3,24_ = 1.02  p = 0.4014 | F_3,24_ = 0.52  p = 0.6735 | F_3,24_ = 0.45  p = 0.7210 |
|  |  |  |  |  |  |
| **Estatus** | F_1,20_ = 3.80  p = 0.0655 | F_1,20_ = 31.64  p = 0.2150 | F_1,20_ = 30.42  p = 0.5237 | **F_1,20_ = 35.46**  **p = 0.0300** | F_1,20_ = 30.18  p = 0.6748 |
|  |  |  |  |  |  |
| **Estatus x Precip** | F_1,20_ = 33.30  p = 0.0843 | F_1,20_ = 30.05  p = 0.8234 | F_1,20_ = 30.79  p = 0.3841 | F_1,20_ = 30.71  p = 0.4096 | F_1,20_ = 30.82  p = 0.3748 |
|  |  |  |  |  |  |
| **TFtype x Estatus** | F_3,20_ = 2.75  p = 0.0694 | F_3,20_ = 0.01  p = 0.9986 | F_3,20_ = 1.90  p = 0.1617 | F_3,20_ = 1.73  p = 0.1940 | **F_3,20_ = 3.64**  **p = 0.0304** |
|  |  |  |  |  |  |
| **TFtype x Estatus x Precip** | F_3,20_ = 0.29  p = 0.8309 | F_3,20_ = 0.53  p = 0.6688 | F_3,20_ = 0.04  p = 0.9869 | F_3,20_ = 0.13  p = 0.9400 | F_3,20_ = 0.73  p = 0.5472 |

† Numerator, denominator degrees of freedom

**Table S3:** Analysis of variance (ANOVA) results for the effects of independent treatment variables on tall fescue biomass and on soil C and N concentrations. Green shoot weights and dead shoot weights were determined by separating total aboveground material (total shoot weight) into live (green) and senesced/dead (brown) portions to record weights for statistical analysis, and then recombined to ball-grind for nutrient analyses. Significant p-values (α = 0.05) are denoted with bold type.

|  | **Root weight** | **Total shoot weight** | **Root: Shoot** | **Green shoot weight** | **Dead shoot weight** | **Soil C** | **Soil N** |
| --- | --- | --- | --- | --- | --- | --- | --- |
| Treatment Effect | **- g -** | **- g -** | **--** | **- g -** | **- g -** | **- % -** | **- % -** |
| **Precip** | F_1,4_^†^ = 0.48  p = 0.5250 | F_1,4_ = 0.10  p = 0.7672 | F_1,4_ = 2.18  p = 0.2141 | F_1,4_ = 0.17  p = 0.7019 | F_1,4_ = 0.01  p = 0.9195 | F_1,4_ = 3.74  p = 0.1254 | F_1,4_ = 2.23  p = 0.2096 |
|  |  |  |  |  |  |  |  |
| **TFtype** | F_3,24_ = 0.91  p = 0.4525 | F_3,24_ = 1.52  p = 0.2353 | F_3,24_ = 2.94  p = 0.0537 | F_3,24_ = 0.75  p = 0.5321 | F_3,24_ = 2.31  p = 0.1014 | F_3,24_ = 1.50  p = 0.2392 | F_3,24_ = 1.14  p = 0.353 |
|  |  |  |  |  |  |  |  |
| **TFtype x Precip** | F_3,24_ = 0.95  p = 0.4327 | F_3,24_ = 1.11  p = 0.3643 | F_3,24_ = 1.97  p = 0.1454 | F_3,24_ = 1.41  p = 0.2637 | F_3,24_ = 0.20  p = 0.8941 | F_3,24_ = 0.34  p = 0.7957 | F_3,24_ = 0.73  p = 0.5455 |
|  |  |  |  |  |  |  |  |
| **Estatus** | **F_1,20_ = 7.45**  **p = 0.0129** | **F_1,20_ = 14.03**  **p = 0.0013** | F_1,20_ = 0.98  p = 0.3333 | **F_1,20_ = 15.49**  **p = 0.0008** | **F_1,20_ = 15.51**  **p = 0.0008** | F_1,20_ = 1.93  p = 0.1796 | F_1,20_ = 3.01  p = 0.0981 |
|  |  |  |  |  |  |  |  |
| **Estatus x Precip** | F_1,20_ = 0.90  p = 0.3544 | **F_1,20_ = 5.63**  **p = 0.0278** | **F_1,20_ = 4.82**  **p = 0.0401** | **F_1,20_ = 5.61**  **p = 0.0281** | **F_1,20_ = 4.99**  **p = 0.0371** | F_1,20_ = 0.00  p = 0.988 | F_1,20_ = 0.00  p = 0.9593 |
|  |  |  |  |  |  |  |  |
| **TFtype x Estatus** | F_3,20_ = 1.72  p = 0.1951 | F_3,20_ = 2.27  p = 0.112 | F_3,20_ = 1.28  p = 0.3083 | F_3,20_ = 1.67  p = 0.2046 | **F_3,20_ = 5.10**  **p = 0.0088** | F_3,20_ = 0.71  p = 0.5589 | F_3,20_ = 0.68  p = 0.5745 |
|  |  |  |  |  |  |  |  |
| **TFtype x Estatus x Precip** | F_3,20_ = 0.81  p = 0.5034 | F_3,20_ = 2.90  p = 0.0606 | F_3,20_ = 2.61  p = 0.0795 | **F_3,20_ = 3.25**  **p = 0.0434** | F_3,20_ = 1.23  p = 0.3239 | F_3,20_ = 1.12  p = 0.3664 | F_3,20_ = 1.35  p = 0.2862 |

† Numerator, denominator degrees of freedom
